# Molecular Mechanisms of AhpC in Resistance to Oxidative Stress in *Burkholderia thailandensis*

**DOI:** 10.3389/fmicb.2019.01483

**Published:** 2019-07-02

**Authors:** Bing Zhang, Huawei Gu, Yantao Yang, Haonan Bai, Chao Zhao, Meiru Si, Tao Su, Xihui Shen

**Affiliations:** ^1^State Key Laboratory of Crop Stress Biology for Arid Areas, College of Life Sciences, Northwest A&F University, Yangling, China; ^2^Shaanxi Key Laboratory of Agricultural and Environmental Microbiology, College of Life Sciences, Northwest A&F University, Yangling, China; ^3^College of Life Sciences, Qufu Normal University, Qufu, China

**Keywords:** alkyl hydroperoxide reductase subunit C, *Burkholderia thailandensis*, oxidative stress, reactive nitrogen species, molecular mechanism

## Abstract

*Burkholderia thailandensis* is a model organism for human pathogens *Burkholderia mallei* and *Burkholderia pseudomallei*. The study of *B. thailandensis* peroxiredoxin is helpful for understanding the survival, pathogenic infection, and antibiotic resistance of its homologous species. Alkyl hydroperoxide reductase subunit C (AhpC) is an important peroxiredoxin involved in oxidative damage defense. Here, we report that *Bth*AhpC exhibits broad specificity for peroxide substrates, including inorganic and organic peroxides and peroxynitrite. AhpC catalyzes the reduction of oxidants using the N-terminal conserved Cys57 as a peroxidatic Cys and the C-terminal conserved Cys171 and Cys173 as resolving Cys. These three conserved Cys residues play critical roles in the catalytic mechanism. AhpD directly interacts with AhpC as an electron donor, and the conserved Cys residues in active site of AhpD are important for AhpC reduction. AhpC is directly repressed by OxyR as shown by identifying the OxyR binding site in the *ahpC* promoter with a DNA binding assay. This work sheds light on the function of AhpC in the peroxides and peroxynitrite damage response in *B. thailandensis* and homologous species.

## Introduction

Obligate aerobes inescapably encounter endogenous sources or exogenous sources reactive oxygen species (ROS) like hydrogen peroxides (H_2_O_2_), alkyl hydroperoxide [ROOH, cumene hydroperoxide (CHP), and *t*-butyl hydroperoxide (*t*-BOOH)], the superoxide anion (${\text{O}}_{2}^{•}-$) and the hydroxyl radical (HO^•^) (Halliwell and Gutteridge, 1984; Chandra et al., 2000). Excess ROS can cause cell damage to lipids, nucleic acids, proteins, or metal cofactors (den Hengst and Buttner, 2008). Enzymatic and non-enzymatic systems are used to counteract ROS toxicity (Liochev, 2013). The peroxiredoxins are large peroxidase family found in most living organisms, and is responsible for antioxidant defense (Guimaraes et al., 2005). Alkyl hydroperoxide reductase subunit C (AhpC) is an important member of peroxiredoxin family that is widely conserved in prokaryotes.

AhpC plays a role in bacterial physiology involving ROS and reactive nitrogen species (RNI) resistance, biofilm formation, survival, persistence, and colonization (Loprasert et al., 2003; Cosgrove et al., 2007; Kimura et al., 2012; Oh and Jeon, 2014). AhpC belongs to the typical 2-Cys peroxiredoxins, important antioxidant enzymes for bacterial defense against oxidative damage caused by ROS. There are two or three conserved cysteine residues critical for enzymatic activity in the AhpC N-terminus and C-terminus (Bryk et al., 2002). AhpCs form a homodimer to catalyze the reduction of peroxides and peroxynitrite via the N-terminal conserved Cys (the peroxidatic Cys, C_P_) to form the intermediate cysteine sulfenic acid (Cys-SOH). A C-terminal conserved Cys (the resolving cysteine, C_R_) on the other AhpC in the dimer attacks the cysteine sulfenic acid to generate a stable disulfide bond and release water (Ellis and Poole, 1997; Guimaraes et al., 2005). In *Mycobacterium tuberculosis*, there is one conserved Cys residue as C_P_ in the N-terminus, and two C_R_ residues in the C-terminus in which another disulphide is to be attacked by external thiol (Wong et al., 2017). To complete the catalytic cycle, the oxidized AhpCs are reduced by alkyl hydroperoxide reductase AhpD, AhpF, or thioredoxin C (TrxC) (Bryk et al., 2002; Jaeger et al., 2004; Koshkin et al., 2004; Wong et al., 2017). In *Mycobacteria*, AhpC is reduced by AhpD instead of AhpF. AhpD recycles AhpC by linking dihydrolipoamide dehydrogenase (Lpd) and dihydrolipoamide succinyltransferase (SucB) to transfer electrons from NADH to AhpC. The study of AhpC is helpful for understanding of bacteria and useful for developing cancer prevention strategies in humans (Guo et al., 2017).

Transcription regulator OxyR plays important roles in redox sensing and protein expression regulation in response to oxidative stress. OxyR has a conserved Helix-Turn-Helix (HTH) DNA binding motif at the N-terminus and an oligomerization motif at the C-terminus, allowing it to bind to the promoter of target genes as a tetramer (Zheng et al., 1998). The reduced OxyR remains inactive and becomes active when oxidized to regulate target gene transcription, upon exposure to oxidative stress (Pomposiello and Demple, 2001). OxyR regulates more than 20 kinds of antioxidant genes like alkyl hydroperoxide reductase (*ahpCF*), hydroperoxidase I (*katG*), glutathione reductase (*gorA*), and glutaredoxin 1 (*grxA*) and others (den Hengst and Buttner, 2008). OxyR was reported to regulate *ahpC* expression in several bacteria species like *E. coli*, *M. marinum*, *M. tuberculosis*, and *S. coelicolor* (Pagan-Ramos et al., 1998; den Hengst and Buttner, 2008). The regulation of *ahpC* expression by OxyR is a field of active research.

*Burkholderia thailandensis* is a non-pathogenic gram-negative bacterium commonly used as a model organism for the related human pathogens *Burkholderia mallei* and *Burkholderia pseudomallei* (Haraga et al., 2008). *B. mallei* and *B. pseudomallei* are potential biological weapons because they can cause the highly contagious even fatal diseases, glanders, and melioidosis (Wiersinga et al., 2006; Saikh and Mott, 2017). *B. thailandensis* is studied as a model organism because of its non-pathogenic characteristics and relatively easy genetic manipulation tools (Garcia, 2017). AhpC is reported to be involved in ROS and RNI defense in *B. pseudomallei* and even in T cell immunity in pathogenic processes (Loprasert et al., 2003; Reynolds et al., 2015). Although many efforts have been made to the research of these bacteria, there are still many areas that must be explored.

We previously reported work on the manganese scavenging and oxidative stress response mediated by type VI secretion system in *B. thailandensis* (Si et al., 2017c). It was found that *ahpC* (BTH_I2092) played a role in protecting against oxidative stress in *B. thailandensis*. Here, we describe the following detailed work on the function of AhpC *in vivo* and *in vitro*. AhpC catalyzes the reduction of hydroperoxides and peroxynitrite *in vitro* and improves the defense activity of *B. thailandensis* against hydroperoxides and peroxynitrite. The conserved Cys^57^ is the peroxidatic cysteine residue (C_P_) that is critical for the AhpC activity like in other homologs. Cys^171^ and Cys^173^ work as resolving cysteine residues (C_R_) that play almost equal roles and could functionally substitute each other. The conserved Cys residues of the electron donor AhpD are important for the reducing AhpC. Thus, AhpC plays an important role in defending *B. thailandensis* from oxidative damages caused by ROS.

## Materials and Methods

### Bacterial Strains and Growth Conditions

All strains and plasmids used in this study are listed in Supplementary Table S1. The *B. thailandensis* E264 derivatives were mutations of the *B. thailandensis* E264 strain (Zhao and Shao, 2015). The *B. thailandensis* strains were cultured on LB plates or in LB medium in a rotary shaker (220 rpm) at 37°C.

### Plasmid Construction

Genes encoding *B. thailandensis* E264 AhpC (BTH_I2092), dihydrolipoamide dehydrogenases Lpd (BTH_I2554), dihydrolipoamide acyltransferase SucB (BTH_I1865), TrxC (BTH_I2218), and thioredoxin reductase TrxR (BTH_I1560) were amplified using PCR with *B. thailandensis* E264 genomic DNA as the template. These DNA fragments were digested using corresponding restriction enzymes and cloned into pET-28a vectors to construct the plasmids pET-28a-*ahpC*, pET-28a-*lpd*, pET-28a-*sucB*, pET-28a-*trxC*, and pET-28a-*trxR*.

To prepare the *ahpC* (BTH_I2092) in-frame deletion mutant of *B. thailandensis*, pDM4-pheS-Δ*ahpC* was constructed. The primer pairs *ahpC*-M1F *Spe*I/ahpC-M1R and *ahpC*-M2F/*ahpC*-M2R *Bgl*II were used to amplify the *ahpC* upstream and downstream fragments with *B. thailandensis* genomic DNA as the PCR template. The primer pair *ahpC*-M1F *Spe*I/*ahpC*-M2R *Bgl*II was used for overlap PCR to fuse the upstream and downstream fragments together. Finally, the PCR product was digested using *Spe*I and *Bgl*II and inserted into pDM4-*phes* to create the plasmid pDM4-*pheS*-Δ*ahpC.* pME6032-*ahpC* was constructed to complement the *ahpC* mutant. The primers *ahpC*-F *Bam*HI and *ahpC*-R *Xho*I were used to amplify the *ahpC* gene from *B. thailandensis* genomic DNA. The *ahpC* gene was digested by *Bam*HI and *Xho*I and ligated into pME6032 and pGEX-6P-1, obtaining the plasmids pME6032-*ahpC* and pGEX-6P-1-*ahpC*, respectively. The point mutant *ahpC*^*C*57*S*^ was created using overlap PCR (Zhang et al., 2013) to substitute a Ser residue for the Cys residue at position 57 of *ahpC*. The primer pairs *ahpC*-F BamHI/*ahpC*^*C*57*S*^ R and *ahpC*^*C*57*S*^ F/*ahpC*-R XhoI were used to amplify the upstream and downstream fragments of *ahpC*. The full-length *ahpC*^*C*57*S*^ was obtained using overlap PCR. The other site-directed mutants such as *ahpC*^*C*171*S*^, *ahpC*^*C*173*S*^, *ahpC*^*C*57*SC*171*S*^, *ahpC*^*C*57*SC*173*S*^, and *ahpC*^*C*171*SC*173*S*^ were created the same way. The *ahpC* and its mutant fragments were digested using *Bam*HI and *Xho*I, and ligated into pET-28a to obtain the expression vectors like pET-28a-*ahpC*. The point mutants *ahpD*^*C*131*S*^ and *ahpD*^*C*134*S*^ were obtained using overlap PCR in the same way. The primer pairs are listed in Supplementary Table S2. *ahpD* (BTH_I2091) and its mutant fragments were then digested using *Bam*HI and *Xho*I, and ligated into pET-21a to obtain the expression vectors pET-21a-*ahpD*, pET-21a-*ahpD*^*C*131*S*^, and pET-21a-*ahpD*^*C*134*S*^.

### In-Frame Deletion and Complementation Mutant Construction in *B. thailandensis*

The in-frame deletion and complementation mutant construction in *B. thailandensis* was performed as described previously (Si et al., 2017c). The pDM4-*pheS* derivatives were transformed into *B. thailandensis* using *E. coli* SM10(λ pir)-mediated conjugational mating to carry out a single crossover. The transconjugants were plated on LB agar with chloramphenicol and streptomycin. The single clones were assayed using PCR. For complementation, the pME6032 derivatives were transformed into the Δ*ahpC* mutant using electroporation. The expression was induced using 1 mM IPTG.

### Sensitivity Assay

A stationary phase strain was diluted 30-fold into M9 medium containing 0.25 mM CHP, 1 mM H_2_O_2_, 1 mM *t*-BOOH, or 20 mM 3-Morpholinosydnonimine (SIN-1) (Aladdin). After 30 min of treatment at 37°C, the culture was diluted to 10^−4^ and plated on LB agar. After 16 h of cultivation at 37°C, the colonies were counted and the survival percentage was calculated as described previously (Wang et al., 2015; Si et al., 2017b).

### Measurement of Intracellular ROS

The fluorescent reporter dye 2′,7′-dichlorodihydrofluorescein diacetate (DCFHDA) (Invitrogen) was used to detect intracellular ROS as previously described (Si et al., 2014, 2017c). 1 mL of culture was collected, washed in PBS and resuspended in 1 mL PBS with 10 μM DCFHDA. The sample was incubated in the dark at 37°C for 40 min, pelleted and washed in PBS twice. 1 mM H_2_O_2_, 0.25 mM CHP, or 0.3 mM *t*-BOOH was added to the sample resuspended in LB and incubated for 30 min at 37°C. The cells were pelleted, washed in PBS and resuspended in 400 μL PBS. 200 μL of the resuspended sample was transferred into a dark 96-well plate. Fluorescence signals were measured using a SpectraMax M2 Plate Reader (Molecular Devices) with excitation/emission wavelengths of 495/520 nm.

### Protein Expression and Purification

To obtain the His_6_-tagged and GST-tagged recombinant proteins, the pET-21a, pET-28a, and pGEX-6P-1 expression vectors were transformed into the *E. coli* BL21(DE3) host strain as described previously (Shen et al., 2005; Si et al., 2014, 2015a). The bacteria were cultured in 500 mL LB medium at 37°C to an OD of about 0.3, cooled to 26°C, and induced to express with 0.5 mM IPTG at 26°C for 8 h. The cell pellet was harvested, resuspended in 30 mL PBS and lysed using ultrasonication. After centrifugation, the supernatant was loaded onto 1 mL pre-equilibrated Ni-NTA (Qiagen) or the GST-Bind resin (Novagen). 5 column volume washing buffer was loaded onto Ni-NTA or GST-Bind resin to wash and elution buffer with 0.5 M imidazole or 10 mM glutathione (GSH) was used to elute the recombinant protein. The His_6_ tag was cleaved by adding 10 units of Enterokinase-Max (Invitrogen, Karlruhe, Germany) and incubating at 4°C overnight to conduct subsequent enzyme activity analysis. Ni-NTA agarose was used to remove the cleaved tag and uncleaved protein from the tag-free protein. All enzymes were purchased from Sigma-Aldrich (St. Louis, MO, United States). The pooled protein was determined using SDS-PAGE to be over 90% homogeneous, and its concentration was determined using Bradford assay according to the manufacturer’s instructions (Bio-Rad) with BSA as the standard.

### Enzymatic Activity Assay of AhpC

As reported previously, Lpd, SucB, AhpD, and AhpC together sustained peroxide-dependent oxidation of NADH. The enzymatic activity of AhpC was determined by monitoring the consumption of NADH at 340 nm (Bryk et al., 2002; Si et al., 2015a,b, 2017a). The Trx reaction system was the same as described previously (Wong et al., 2017). The reaction mixture contained 100 mM KPi (pH 7.0), 1 mM EDTA, 150 μM NADH, 1 μM AhpC, 40 μM AhpD, 2 μM SucB, 2 μM Lpd and peroxides (1 mM H_2_O_2_ or 0.5 mM *t*-BOOH or CHP); or contained 50 mM Tris–HCl buffer (pH 8.0), 2 mM EDTA, 250 μM NADPH, 1 μM AhpC, 1 mM peroxides, and the reduced Trx-generating system [5 μM thioredoxin reductase (TrxR) and 40 μM TrxC]. After 5 min reaction, peroxides were added into the reaction mixture and then the NADH or NADPH consumption was monitored by the absorbance at 340 nm. The catalytic kinetic parameters for one substrate were acquired by changing its concentration in the case of saturating concentrations of the other substrate (between 0 and 500 μM for AhpD or TrxC, and between 0 and 1 mM for peroxide). The activity was determined after subtracting the spontaneous reduction rate detected without AhpC, and the micromoles amount of NADPH or NADH oxidized per second per micromolar of enzyme (i.e., turnover number, s^−1^) was calculated using the molar absorption coefficient of NADPH or NADH at 340 nm (ε_340_) of 6220 M^−1^ cm^−1^. Three independent experiments were performed at each substrate concentration. The *k*_cat_ and *K*_m_ values of AhpC were acquired from a non-linear fit to the Michaelis–Menten equation using the program GraphPad Prism 5.

### Determination of the Peroxynitrite Reductase Activity of AhpC

The reaction rate of AhpC with peroxynitrite was examined using a competition approach by measuring the inhibitor effect of increasing AhpC concentrations on peroxynitrite-mediated HRP oxidation to compound I in a stopped-flow spectrophotometer (Applied Photophysics Ltd., United Kingdom) under our experimental conditions (100 mM phosphate buffer containing 0.1 mM DTPA, pH 7.4, and 25 ± 0.2°C) as previously described (Hayashi and Yamazaki, 1979; Floris et al., 1993; Ogusucu et al., 2007; Trujillo et al., 2008; Hugo et al., 2009; Manta et al., 2009; Demicheli et al., 2016; Reyes et al., 2016). In brief, 1 μM peroxynitrite-mediated HRP (5 μM) oxidation to Compound I in the absence or presence of increasing reduced AhpC concentrations (from 0 to 10 μM) was evaluated at 398 nm (Δε_398_ = 4.2 × 10^4^ M^−1^ cm^−1^). The rate constant of peroxynitrite-mediated AhpC oxidation was calculated from HRP-Compound I yield obtained at different AhpC concentrations (0–10 μM) as previously described (Ogusucu et al., 2007; Hugo et al., 2009; Manta et al., 2009).

### Protein Sulfenic Acid Analysis

To analyze the reaction intermediate Cys-SOH content, NBD chloride was used to label the site-directed mutant AhpC^C57SC171S^, AhpC^C57SC173S^, and AhpC^C171SC173S^ as previously described (Baker and Poole, 2003; Si et al., 2015c). Each mutant protein (50 μM) was incubated with 5 mM DTT for 30 min. The redundant DTT was removed by ultrafiltration. The protein sample was then made anaerobic by flushing with argon gas for 30 min and separated into two parts. One was treated with 50 μM anaerobic CHP and the other kept as a control. The sample was incubated with 5 mM anaerobic NBD chloride for 30 min at 26°C in the dark. After removing excess NBD chloride by ultrafiltration, the protein sample was analyzed spectrophotometrically at 200–600 nm.

### Protein Thiol Content Analysis

Free sulfhydryl groups in AhpC variants were determined using 5, 5′-dithio-bis (2-nitrobenzoic acid) (DTNB) as previously described (Ellman, 1959; Si et al., 2014, 2015c, 2017c). Proteins (25 μM) were treated with 5 mM CHP or 50 mM DTT at 26°C for 30 min. DTT or CHP was removed by ultrafiltration. The resulting proteins (10 μM) were added to DTNB (2 mM) in 50 mM Tris–HCl buffer (pH8.0) and the absorbance at 412 nm was measured against a 2 mM DTNB solution as the reference. The amounts of reactive sulfhydryl groups were determined using the molar absorption coefficient of TNB at 412 nm of 13,600 M^−1^s^−1^ (Eyer et al., 2003).

### Electrophoretic Mobility Shift Assay (EMSA)

Electrophoretic mobility shift assay was performed using the method by Si et al. (2017c). To reduce non-specific binding in the EMSA assay, the *ahpC* promoter region (P*_ahpC_*, 231 bp) containing the predicted OxyR binding site was amplified from the *ahpC* promoter region using primers P*_ahpC_*-F/P*_ahpC_*-R. Increasing concentrations of purified OxyR (0–16 μg) are incubated with 20 ng *ahpC* DNA promoter in EMSA buffer containing 20 mM Tris–HCl, pH7.4, 4 mM MgCl_2_, 100 mM NaCl, 1 mM DTT, and 10% glycerol. The binding reaction mixture is incubated at room temperature for 30 min, subjected to electrophoresis on a 6% native polyacrylamide gel with 5% glycerol in 0.5 × TBE electrophoresis buffer, and detected using SYBR Green. A 230 bp fragment from the *ahpC* coding region amplified with primers Control-F and Control-R instead of the 231 bp *ahpC* promoter in the binding assays was used as a negative control.

### GST Pull-Down Assay

The GST pull-down assay was performed as previously described with minor modifications (Xu et al., 2014; Si et al., 2017b). Briefly, purified GST-AhpC was mixed with His_6_-AhpD in PBS on a rotator for 2 h at 4°C, and GST was used as a negative control. After adding 30 μL of prewashed glutathione beads slurry, binding proceeded for another 2 h at 4°C. The beads were then washed five times with TEN buffer (50 mM Tris–HCl, pH 8.0, 500 mM NaCl). Retained proteins were detected using immunoblot after SDS-PAGE with the anti-His antibody (Millipore).

### Statistical Analysis

Student’s *t*-test was used to test for statistical significance. Statistical analyses were performed using GraphPad Prism Software (GraphPad Software, San Diego, CA, United States).

## Results

### The Antioxidant Function of AhpC in *B. thailandensis*

**FIGURE 1:**
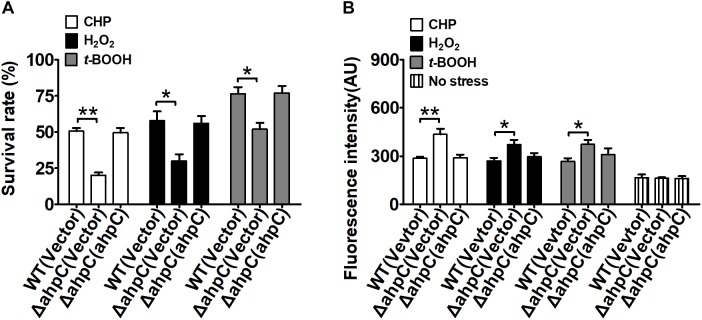
*ahpC* was required for cellular resistance to toxic agent-induced stresses. **(A)** The *B. thailandensis* WT(Vector), Δ*ahpC*(Vector), and Δ*ahpC*(*ahpC*) strains were exposed to H_2_O_2_ (1 mM), CHP (0.25 mM), and *t*-BOOH (0.3 mM) at 37°C for 30 min. The mutant lacking *ahpC* was sensitive to oxidative stress. Cell survival percentage was measured by viability assay. **(B)** The *B. thailandensis* WT(Vector), Δ*ahpC*(Vector), and Δ*ahpC*(*ahpC*) strains were exposed to H_2_O_2_ (1 mM), CHP (0.25 mM), and *t*-BOOH (0.3 mM). The ROS levels in *B. thailandensis* were measured using the DCF fluorescence determination assay after exposure to H_2_O_2_ (1 mM), CHP (0.25 mM), and *t*-BOOH (0.3 mM). The asterisk indicates a significant difference (^∗^*p* < 0.05; ^∗∗^*p* < 0.01, using the Student *t*-test).

To examine the antioxidant functions of AhpC in *B. thailandensis*, the sensitivities of the wild type, the Δ*ahpC* mutant, and the complementary strain Δ*ahpC*(*ahpC*) to CHP (0.25 mM), H_2_O_2_ (1 mM), and *t*-BOOH (0.3 mM) were tested (Figure 1A). Concentrations of peroxides applied could reduce the survival rate of the wild type and increase the mortality rate from 50 to 70% (Supplementary Figure S1A). Although deletion of *ahpC* did not affect bacterial growth under normal conditions (Supplementary Figure S1B), the survival rates of the Δ*ahpC* mutant decreased by about 31.4–52.6% compared to wild type. However, the oxidative stress sensitive phenotype of the Δ*ahpC* mutant recovered to the wild type level after complementation with the *ahpC* gene. In addition, deletion of *ahpC* was shown to affect the survival rate of *B. thailandensis* in response to peroxynitrite (Supplementary Figure S1C).

Excess intracellular ROS is harmful in bacteria. To investigate the function of AhpC in reducing ROS under oxidative stress, the intracellular ROS levels were determined using the membrane-permeable fluorogenic probe 2′,7′-dichlorodihydrofluorescein diacetate (DCFHDA). As shown in Figure 1B, the ROS levels of all three strains under oxidative stress were higher than those of the unstressed strains, indicating ROS induction by oxidative stress in the strains. Moreover, the Δ*ahpC* mutant had a significantly higher ROS level than the wild type after oxidative stress treatment. However, the ROS level of the Δ*ahpC* mutant was completely restored to that of the wild type by complementation (Figure 1B). These results indicate that AhpC plays important roles in counteracting oxidative stress by reducing ROS in *B. thailandensis*.

### The Peroxide Reductase Activity of AhpC *in vitro*

It was reported that AhpC metabolize peroxides and peroxynitrite via a conserved N terminal cysteine residue that is oxidized to form a disulfide bond. To complete the catalytic cycle, the Cys residue must be reduced. AhpC can be regenerated by the reducing agents Trxc and AhpD. Thus, to examine whether the *in vitro* peroxide reductase activity of *B. thailandensis* AhpC was supported by the AhpD system (Lpd, SucB, AhpD, and NADH) and Trx system (TrxC, TrxR, and NADPH), we measured the catalytic constants of *B. thailandensis* AhpC with TrxC or AhpD as recycling reductants under steady-state conditions at saturating concentrations of peroxides (1 mM) and varying concentrations of reductants (0–500 μM). As shown in Table 1, the apparent affinity of *B. thailandensis* AhpC toward AhpD obtained under steady-state conditions was, higher than the value determined with TrxC. The *k*_cat_ values of AhpC-mediated CHP reduction with the TrxC and AhpD systems were 11.6 ± 0.2 s^−1^ and 29.7 ± 2 s^−1^, respectively; and the respective *K*_m_ values were 147.3 ± 10 μM and 69.2 ± 4.1 μM, respectively. The catalytic efficiencies were 0.79 ± 0.2 × 10^5^ M^−1^ s^−1^ and 4.29 ± 1.2 × 10^5^M^−1^ s^−1^, respectively. So AhpD and TrxC were active with an apparent efficiency order AhpD > TrxC. Based on our biochemical studies of AhpC, we speculated that AhpC-dependent peroxide defense was supported primarily by NADH because of the preferred selectivity of AhpD for this cofactor. Moreover, AhpC showed a small preference for the larger hydroperoxides.

**Table 1:** Kinetic constants for different AhpC reducing systems.

Systems	Substrates	*K*_m_ (μM)	*k*_cat_ (s^−1^)	*k*_cat_/*K*_m_ (×10^5^ M^−1^s^−1^)
TrxC	H_2_O_2_	347.9 ± 9	3.6 ± 0.4	0.10 ± 0.04
	*t*-BOOH	202.5 ± 4.7	9.5 ± 0.8	0.47 ± 0.1
	CHP	147.3 ± 10	11.6 ± 0.2	0.79 ± 0.2
AhpD	H_2_O_2_	193.4 ± 8.7	7.1 ± 0.3	0.36 ± 0.1
	*t*-BOOH	97.8 ± 3.5	26.1 ± 1.9	2.67 ± 0.3
	CHP	69.2 ± 4.1	29.7 ± 2.0	4.29 ± 1.2

*Peroxidase assays were performed as described in section “Materials and Methods” with a fixed concentration of peroxides (1 mM) and AhpC (1 μM) using different concentrations of reducing systems: TrxC (0–500 μM) and AhpD (0–500 μM). The data were presented as means of values obtained from three independent assays.*

**FIGURE 2:**
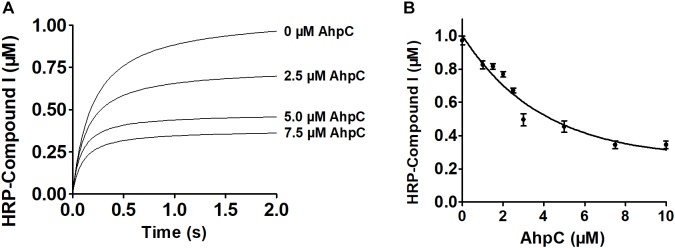
Kinetics of peroxynitrite reduction by AhpC. **(A)** Peroxynitrite (1 μM) in 10 mM NaOH was rapidly mixed with HRP (5 μM) with or without increasing concentrations of AhpC (0.0, 2.5, 5.0, 7.5 μM) in 100 mM sodium phosphate buffer (pH 7.4) at 25°C using a SX20 Stopped-Flow Spectrometer. **(B)** HRP-Compound I concentration formed was plotted compared to AhpC concentration. The continuous line represented HRP-Compound I yields simulated according to a simple competition system using the program GraphPad Prism 5, and the rate constant of AhpC oxidation was calculated.

Next, enzymatic activities of *B. thailandensis* AhpC toward various oxidizing substrates were analyzed using the TrxC and AhpD system as the reducing power (Table 2). Results showed that *B. thailandensis* AhpC had strong reductase activity in treating peroxide H_2_O_2_, *t*-BOOH and CHP (the catalytic efficiencies *k*_cat_*/K*_m_ in AhpD system were 0.31 ± 0.05 × 10^5^ M^−1^s^−1^, 4.24 ± 0.9 × 10^5^ M^−1^s^−1^, and 5.19 ± 1.5 × 10^5^ M^−1^s^−1^, respectively; 0.1 ± 0.03 × 10^5^ M^−1^ s^−1^, 0.91 ± 0.1 × 10^5^ M^−1^ s^−1^, and 1.42 ± 0.2 × 10^5^ M^−1^ s^−1^in TrxC system, respectively). The relatively high reactivity of AhpC with multiple substrates, at 10^5^∼10^6^ M^−1^ s^−1^, supports the view that it acts as a potent antioxidant defense across a range of peroxide substrates. Apart from these peroxides, peroxynitrite was also reported as the substrate of AhpC in *M. tuberculosis*, *S. typhimurium*, and *H. pylori* (Bryk et al., 2000). The capacity of AhpC to reduce peroxynitrite was analyzed using competition approaches as described (Ogusucu et al., 2007). 1 μM peroxynitrite in 10 mM NaOH was mixed with 5 μM HRP in the absence of or with varying concentrations of AhpC in 100 mM sodium phosphate buffer (pH 7.4) at 25°C. As shown in Figure 2A, mixing HRP (5 μM) with peroxynitrite (1 μM) without AhpC led to the formation of HRP-Compound I. However, addition of AhpC caused a decrease in HRP-Compound I formation yield. Kinetic analysis of the data indicated that the second order rate constant of peroxynitrite reduction by AhpC was (2.7 ± 0.53) × 10^6^ M^−1^ s^−1^ at pH 7.4 and 25°C (Figure 2B), which was similar to those reported for other AhpCs (Bryk et al., 2000) Nature, or Prxs (Floris et al., 1993; Ogusucu et al., 2007; Manta et al., 2009; Reyes et al., 2016) in stopped-flow experiments.

**Table 2:** AhpC steady-state kinetic constants for peroxide substrates.

Substrates	Systems					
	AhpD			TrxC		
	*K*_m_ (μM)	*k*_cat_ (s^−1^)	*k*_cat_/*K*_m_ (×10^5^ M^−1^ s^−1^)	*K*_m_ (μM)	*k*_cat_ (s^−1^)	*k*_cat_/*K*_m_ (×10^5^ M^−1^ s^−1^)
H_2_O_2_	238.9 ± 8.6	7.4 ± 0.3	0.31 ± 0.05	321.6 ± 13.2	3.3 ± 0.5	0.1 ± 0.03
*t*-BOOH	60.1 ± 1.8	25.5 ± 0.6	4.24 ± 0.9	107.3 ± 8.1	9.8 ± 0.6	0.91 ± 0.1
CHP	57.6 ± 1.2	29.9 ± 0.4	5.19 ± 1.5	86.8 ± 6.7	12.4 ± 1.1	1.42 ± 0.2

*Peroxidase assays were performed as described in section “Materials and Methods” with a fixed concentration of the reducing systems (40 μM AhpD, 2 μM SucB, and 2 μM Lpd, or 5 μM TrxR and 40 μM TrxC) and AhpC (1 μM) using different concentrations of substrates: 0–1 mM H_2_O_2_, and 0–0.5 mM t-BOOH or CHP. The data were presented as means of values obtained from three independent assays.*

### The Function of Conserved Cysteine Residues in AhpC

**FIGURE 3:**
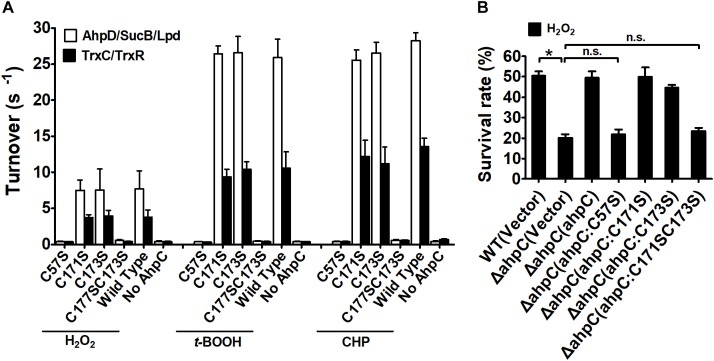
Analysis of the properties of AhpC point mutants. **(A)** Peroxidase activity of AhpC and the mutants in conserved Cys residues examined by AhpD/SucB/Lpd reaction system and TrxC reaction system. Peroxidase assays were performed as described in the section “Materials and Methods” using a fixed concentration of the reducing systems (0–500 μM AhpD, 2 μM SucB, and 2 μM Lpd, or 5 μM TrxR, and 0–500 μM TrxC), AhpC (WT and its variants 1 μM), and substrates (1 mM). **(B)** The *B. thailandensis* WT(Vector), Δ*ahpC*(Vector), Δ*ahpC*(*ahpC*), Δ*ahpC*(*ahpC*^*C*57*S*^), Δ*ahpC*(*ahpC*^*C*171*S*^), Δ*ahpC*(*ahpC*^*C*173*S*^), and Δ*ahpC*(*ahpC*^*C*171*SC*173*S*^) strains were exposed to H_2_O_2_ (1 mM) at 37°C for 30 min. The asterisk indicates a significant difference (^∗^*p* < 0.05; n.s., no significant difference using the Student *t*-test).

Typical peroxiredoxin protein family members possess two conserved Cys residues, and mycobacterial AhpCs employ three Cys residues involved in catalysis (Guimaraes et al., 2005). The amino acid sequence alignment of AhpC homologs indicated that *B. thailandensis* AhpC possesses three conserved Cys residues: Cys^57^, Cys^171^, and Cys^173^. To investigate the function of Cys in AhpC, the site-directed mutations AhpC^C57S^, AhpC^C171S^, AhpC^C173S^, AhpC^C171SC173S^ were produced, which substituted Ser residue for a Cys residue. The peroxidase activities of these proteins were measured in the presence of H_2_O_2_, *t*-BOOH and CHP. As shown in Figure 3A, the single mutant of Cys^57^ or double mutant of Cys^171^ and Cys^173^ resulted in a loss of AhpC catalytic activity. However, single mutant of Cys^171^ or Cys^173^ showed almost no obvious effect on the activity. Similar results were also observed when the Trx system was used as a reducing power. The result indicated that AhpC could be reduced by both AhpD and TrxC, and Cys^171^ and Cys^173^ could substitute each other in function. To examine the intracellular function of conserved Cys residues in AhpC, the sensitivity assay of *B. thailandensis* strains with *ahpC* in-frame deletion or serial mutant complementation were performed using H_2_O_2_ treatment. The result confirmed that *ahpC*^*C*57*S*^ or *ahpC*^*C*57*SC*171*S*^ mutant gene complementation almost could not increase the survival rate of the respective strains, but the *ahpC*^*C*57*S*^ or *ahpC*^*C*57*S*^ complementation could nearly recover the survival rate (Figure 3B).

These results indicate that the conserved Cys^57^ in the N-terminus domain plays a critical role in the catalytic activity of AhpC in *B. thailandensis* like in other bacteria (Wong et al., 2017). The C-terminal Cys^171^ and Cys^173^ residues play important roles and they could functionally compensate for each other. For simplicity, we used the AhpD system only as the reducing power in the following experiments.

### The Mechanism of Conserved Cys in AhpC Catalytic Activity

**FIGURE 4:**
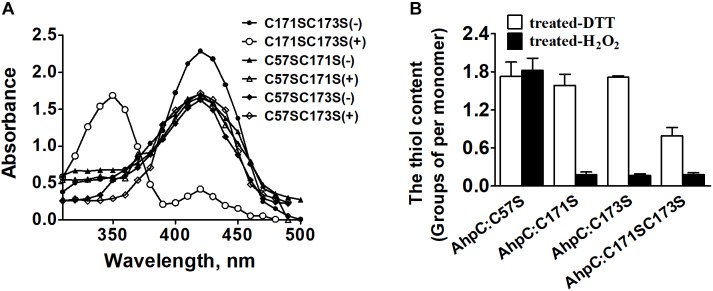
The thiol content and formation of H_2_O_2_-treated AhpC. **(A)** Spectrophotometric analysis of NBD-labeled AhpC variants. Proteins treated with (+) and without (−) H_2_O_2_ were modified with NBD chloride for 30 min. After removal of excess reagent by ultrafiltration, the labeled proteins were analyzed spectrophotometrically at 200–600 nm. **(B)** Free sulfhydryl groups in AhpC variants determined using 5, 5′-dithio-bio (2-nitrobenzoic acid).

The peroxidate cysteine catalyzes the peroxidase reaction by temporarily producing sulfenic acids (Ellis and Poole, 1997). To investigate the functional difference between N-terminal cysteine and C-terminal cysteines in detail, AhpC^C171SC173S^, AhpC^C57SC171S^, and AhpC^C57SC173S^ variants were generated and treated with NBD-Cl (4-chloro-7-nitrobenzo-2-oxa-1,3-diazole) with or without CHP. NBD-Cl can react with the thiol group and sulfenic acids. The reaction between NBD-Cl and the thiol group can generate absorption at 420 nm, and the reaction with sulfenic acids increases absorption at 347 nm (Baker and Poole, 2003). After the reaction with CHP, the AhpC^C171SC173S^ variant exhibited Soret bands at 347 and 420 nm differing from the CHP untreated group. There was no peak change with or without CHP in the reaction of AhpC^C57SC171S^ and AhpC^C57SC173S^ variants (Figure 4A). The results suggest that Cys^57^ works as a peroxidatic cysteine in the active center of AhpC. The quantification of free thiol levels of AhpC variants was also performed with or without H_2_O_2_ treatment (Figure 4B). AhpC^C57S^ showed two thiol groups per monomer with or without H_2_O_2_ treatment. But AhpC^C171S^ and AhpC^C173S^ lost two thiol groups, and AhpC^C171SC173S^ lost one. These data indicate that the N-terminal Cys^57^ residue acts as a peroxidatic cysteine and the C-terminal Cys^171^ and Cys^173^ residues act as resolving cysteines like in other AhpC homologs (Guimaraes et al., 2005).

### C-Terminal Signature Motif Cys-X-X-Cys of AhpD Is Critical for the Catalytic Reaction With AhpC

**FIGURE 5:**
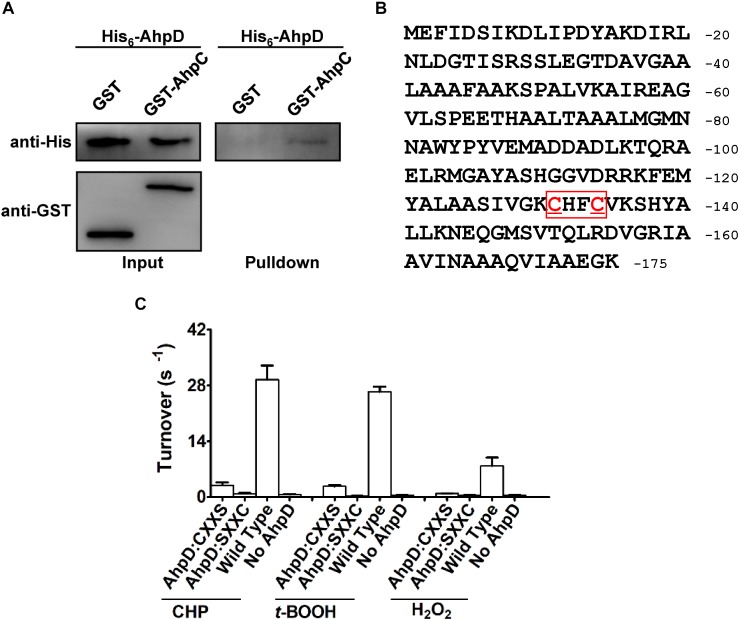
Interaction mechanism between AhpC and AhpD. **(A)** Direct binding of AphC to AphD. His6-AphD was incubated with GST-AphC or GST, and the protein complexes captured with glutathione beads were detected using western blotting. **(B)** Cys-X-X-Cys motif sequence in AhpD. **(C)** Cys in AhpD is required for AhpC’s peroxidase activity. Peroxidase assays were performed as described in section “Materials and Methods” with peroxides (1 mM), AhpC (1 μM), 2 μM SucB, 2 μM Lpd, and AhpD (WT or its variants for 0–500 μM). The data are presented as means of values obtained from three independent assays. Mean values with standard deviations (error bars) from at least three repeats are shown.

In the AhpC/AhpD/SucB/Lpd peroxide reducing reaction system, AhpD is the electron donor for AhpC, which is important for the reaction. To test the direct interaction between AhpD and AhpC, the GST pulldown assay was performed as shown in Figure 5A. The results showed direct binding of AhpD to AhpC. AhpD possesses a signature motif at the C-terminus as Cys-X-X-Cys which is critical for the catalytic activity in *M. tuberculosis* (Bryk et al., 2002). To test the function of AhpD and the Cys-X-X-Cys motif (Figure 5B), Cys to Ser mutant proteins in the motif were designed and purified. AhpD and the variants were used to carry out the reductase assay with AhpC/SucB/Lpd against CHP, *t*-BOOH, H_2_O_2_ and peroxynitrite. The activities of AhpD^CXXS^ decreased to about 10% of wild type, and the activities of AhpD^SXXC^ nearly disappeared (Figure 5C). The results indicated that Cys^131^ is critical for AhpD the reductase activity, and Cys^134^ also had an important influence on the enzyme activity that differs from AhpD in *M. tuberculosis* (Koshkin et al., 2004). The Cys-X-X-Cys motif of AhpD plays a critical role in the peroxide and peroxynitrite reducing reaction with AhpC. As such, AhpD functions as a dithiol mixed disulfide reductase with the active site cysteines.

### The Regulation of AhpC by OxyR in *B. thailandensis*

**FIGURE 6:**
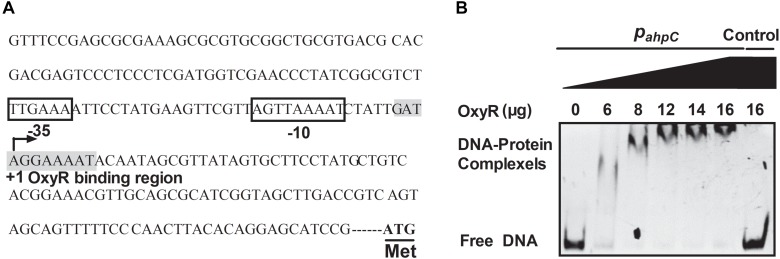
OxyR directly binds to the AhpC promoter. **(A)** *ahpC* promoter structure analysis. ATG was the starting codon. The shadow part was the binding region of OxyR. The binding regions of OxyR were labeled by square box. +1: The transcriptional starting point. **(B)** Gel migration experiment verified the binding of OxyR to *ahpC* promoter. Gradient concentration (0, 6, 8, 12, 14, 16 μg) of OxyR were mixed with P*_ahpC_* (*ahpC* primer DNA containing putative OxyR binding region) and separated by EMSA. DNA without the OxyR binding region was mixed with OxyR as control.

The OxyR protein is a well-known regulator for oxidative stress in bacteria. In our previous study, the negative regulation of AhpC by OxyR was discovered using RNA sequencing (RNA-seq)-based transcriptomic analysis and quantitative reverse transcriptase PCR (qRT-PCR) (Si et al., 2017c). To explore the regulation between OxyR and the expression of *ahpC*, promoter sequence analysis was performed and an OxyR binding site in the promoter region of *ahpC* was identified using the online software Virtual Footprint ^1^ (Figure 6A). To examine the direct interaction between OxyR and *ahpC* promoter DNA, a promoter region DNA fragment containing the putative OxyR binding site was amplified using PCR with P*_ahpC_*-F/P*_ahpC_*-R primer pair was incubated with varying concentrations of purified OxyR protein *in vitro*. The reaction mixture was analyzed using an EMSA. As shown in Figure 6B, OxyR directly bound to the *ahpC* promoter region DNA fragment, but the negative control unrelated DNA without the OxyR binding site showed no interaction with OxyR. These data provided direct evidence that OxyR binds to the promoter region of *ahpC* to regulate its expression.

## Discussion

Reactive oxygen species are involved in aerobic metabolism in living organisms. In addition to causing harmful stresses in plant and animal, ROS also play a role as signaling during stress defense and aging (Apel and Hirt, 2004; Liochev, 2013; Moloney and Cotter, 2018). In bacteria, ROS play important roles in host-pathogen interactions, infection and antibiotic resistance (Marrakchi et al., 2014; Paiva and Bozza, 2014). Abnormal ROS levels activate a regulator, like OxyR and OhrR, and increase the expression and activity of peroxidase for antioxidant defense AhpC or KatG (Pagan-Ramos et al., 1998). The study of the function and catalytic mechanism of peroxidase AhpC is helpful for understanding the mechanism of bacterial survival, infection and antibiotic resistance.

AhpC was confirmed to play a critical role in *B. thailandensis* oxidant defense. The deletion of the *ahpC* gene in *B. thailandensis* caused an ROS level increase and a survival rate decrease *in vivo* after the exposure of the cells to peroxides. *ahpC* gene complementation could recover the ROS level and survival rate (Figure 1). The AhpC activity *in vitro* also showed its function in oxidant defense like in other bacteria including *E. coli*, *M. tuberculosi*s, and *M. marinum* (Pagan-Ramos et al., 1998; Ritz et al., 2001; Seaver and Imlay, 2001). AhpC in *B. thailandensis* can also catalyze the conversion of peroxynitrite to nitrite as reported in *M. tuberculosis*, *S. typhimurium*, *M. tuberculosis*, and *H. pylori* (Bryk et al., 2000). The difference between the activities against peroxides and peroxynitrite of AhpC may reflect the partial functional complementation of KatG, SOD, and other antioxidant enzymes indicating various antioxidant mechanisms in living organisms.

AhpC of *B. thailandensis* is a 2-Cys peroxiredoxin and the conserved Cys residues are important for its function. Cys^57^ was proved to be the peroxidatic Cys residue that was critical for the function of AhpC (Figure 3, 4). Cys^171^ and Cys^173^ were both recognized as resolving Cys residues and together play important roles like the resolving Cys residues of *Mtb*AhpC and *Mb*AhpC (Guimaraes et al., 2005; Wong et al., 2017). Cys^171^ and Cys^173^ worked nearly equally as resolving residues. However, the survival rate of *ahpC*^*C*171*S*^ complementation was higher than *ahpC*^*C*173*S*^ indicating that Cys^173^ plays a more important role than Cys^171^. The apparent functions of these two Cys residues differ from the resolving Cys residues in *Mtb*AhpC, wherein Cys^176^ could only substitute for Cys^174^ to some extent (Koshkin et al., 2004). To explore the reason for the difference, detailed structural biology data are needed for *Bth*AhpC.

The oxidized AhpC was reported to be reduced by AhpD, AhpF, TrxB, or TrxC in bacteria. AhpC is reduced by AhpD and TrxC instead of AhpF and TrxB in mycobacteria, respectively (den Hengst and Buttner, 2008). Here we showed that AhpC can be reduced by AhpD and TrxC in *B. thailandensis* like in *M. tuberculosis* (Figure 3A). As an electron donor, AhpD reduces AhpC via the Cys-X-X-Cys motif in the active site (Bryk et al., 2002). In the *Mtb*AhpD active site, Cys^133^ but not Cys^130^ directly interacts with Cys^61^ of AhpC to form a disulfide cross-link, because Cys^133^ lies at the base of active site cleft and easily interacts with other molecules while Cys^130^ is partially buried in the fold (Bryk et al., 2002; Koshkin et al., 2004). In *B. thailandensis*, the Cys-X-X-Cys motif of AhpD plays a critical role in reducing AhpC, and Cys^131^ had a much more important role than Cys^134^ (Figure 5). The *Bth*AhpD active site Cys-X-X-Cys motif has a similar function mechanism to *Mtb*AhpD but it may differ in structure. More effort in the structural biology of *Bth*AhpD is needed to illuminate the mechanism in future.

The *ahpC* gene is negatively regulated by OxyR in *B. thailandensis* based on detection of the transcriptional level of *ahpC* in an *oxyR*-null mutant strain (Si et al., 2017c). The OxyR binding site in the *ahpC* promoter was identified and shown using EMSA to be especially bound by OxyR (Figure 6). The OxyR binding site in the *ahpC* promoter of *B. thailandensis* has a sequence signature like the sequences in other bacteria such as *N. meningitidis*, *M. tuberculosis*, *M. marinum*, *M. leprae*, and some other *mycobacterium* (Pagan-Ramos et al., 1998; Ieva et al., 2008). OxyR negatively regulates AhpC in *B. thailandensis* differently than in *E. coli*, *S. coelicolor* and *M. marinum*, where OxyR activates *ahpC* gene expression (den Hengst and Buttner, 2008), and similarly to its negative regulator activity for catalase in *N. gonorrhoeae* and *C. diphtheriae* (Tseng et al., 2003; Kim and Holmes, 2012). The inducing regulation of AhpC by OxyR is important for the bacteria’s oxidation stress response.

In conclusion, this study reveals that AhpC plays an important role in peroxide and peroxynitrite defense in *B. thailandensis*. AhpC catalyzes the reducing activity via the three conserved Cys residues and AhpC reduction is affected by the conserved Cys residues in the active site of AhpD.

## Data Availability

The raw data supporting the conclusions of this manuscript will be made available by the authors, without undue reservation, to any qualified researcher.

## Author Contributions

BZ and HG contributed equally to the design of the study and the experiments. HB and CZ performed the statistical analysis. YY wrote the first draft of the manuscript. HG and MS wrote the sections of the manuscript. XS and TS designed the study and are responsible for this work. All authors contributed to the revision, read and approved the final version of the manuscript for submission.

## Conflict of Interest Statement

The authors declare that the research was conducted in the absence of any commercial or financial relationships that could be construed as a potential conflict of interest.

## Footnotes

1 www.prodoric.de/vfp

## Supplementary Material

FIGURE S1 The growth conditions of *B. thailandensis* and variants in cultivation with or without oxidants. **(A)** The survival rate of *B. thailandensis* against different concentrations of peroxides. **(B)** The growth curve of *B. thailandensis* and variant without oxidant. **(C)** The survival rate of *B. thailandensis* and variants against 20 mM SIN-1. The asterisk indicates a significant difference (^∗^*p* < 0.05, using the Student *t*-test).

TABLE S1 Bacterial strains and plasmids used in this study.

TABLE S2 Primers used in this study.
