# Supplementary figures and images for: Molecular Mechanisms of AhpC in Resistance to Oxidative Stress in Burkholderia thailandensis
Source: Front Microbiol. 2019 Jul 2;10:1483. doi: 10.3389/fmicb.2019.01483 (PMC6626918; doi:10.3389/fmicb.2019.01483)

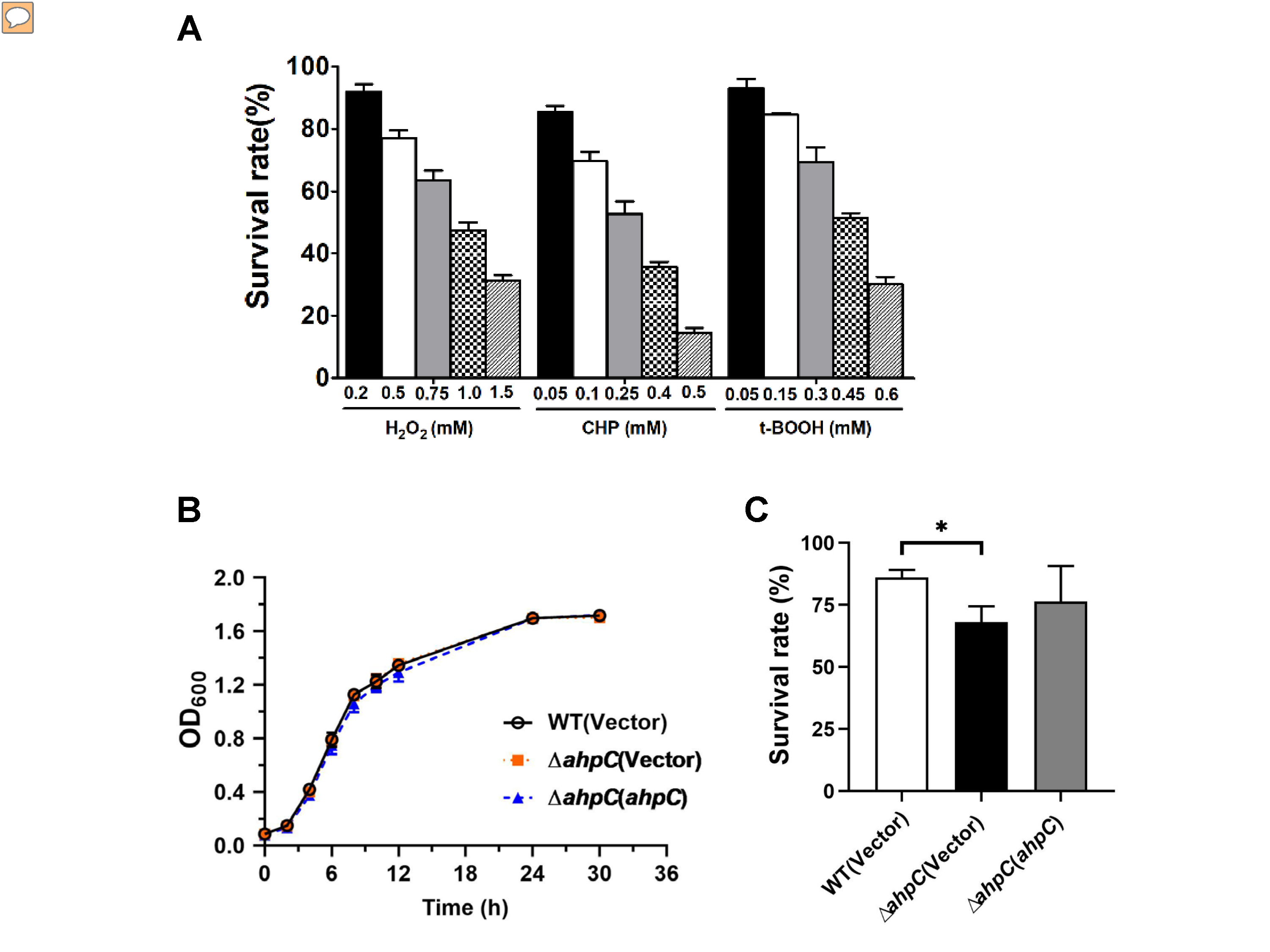

Supplement: FIGURE S1 — The growth conditions of B. thailandensis and variants in cultivation with or without oxidants. (A) The survival rate of B. thailandensis against different concentrations of peroxides. (B) The growth curve of B. thailandensis and variant without oxidant. (C) The survival rate of B. thailandensis and variants against 20 mM SIN-1. The asterisk indicates a significant difference (∗p < 0.05, using the Student t-test). [file Image_1.TIF]
