# Supplementary material for: Molecular Mechanisms of AhpC in Resistance to Oxidative Stress in Burkholderia thailandensis
Source: Front Microbiol. 2019 Jul 2;10:1483. doi: 10.3389/fmicb.2019.01483 (PMC6626918; doi:10.3389/fmicb.2019.01483)
Supplement: TABLE S1 — Bacterial strains and plasmids used in this study. [file Table_1.DOCX]

Table S1. Bacterial strains and plasmids used in this study

| Strains or plasmids | Relevant genotype description | References |
| --- | --- | --- |
| ***E. coli*** |  |  |
| BL21(DE3) | Host for expression vector pET15b, pET21a and pET28a | Novagen |
| JM109 | Host for cloning | Stratagene |
| SM10 λ pir | λ-pir lysogen of SM10, *thi pro hsdR hsdM^+^* *recA* RP4 2-Tc::Mu-Km::Tn7 | (Si et al., 2017) |
| ***Burkholderia thailandensis*** |  |  |
| E264 | Wild type strain (ATCC 700388) environmental isolate from Thailand Str^r^ | (Si et al., 2017) |
| Δ*ahpC* | *ahpC* (*BTH_I2092*) gene deleted in *B. thailandensis E264*, Str^r^ | This study |
| WT(Vector) | Wild type containing pME6032 vector, Str^r^, Tc^r^ | This study |
| Δ*ahpC*(Vector) | Δ*ahpC* mutant containing pME6032 vector, Str^r^, Tc^r^ | This study |
| Δ*ahpC*(*ahpC*) | Complementation of *ahpC* in Δ*ahpC*, Str^r^, Tc^r^ | This study |
| *ΔahpC*(*ahpC^C57S^*) | Complementation of *ahpC^C57S^* in Δ*ahpC*, Str^r^, Tc^r^ | This study |
| Δ*ahpC*(*ahpC^C171S^*) | Complementation of *ahpC^C171S^* in Δ*ahpC*, Str^r^, Tc^r^ | This study |
| Δ*ahpC*(*ahpC^C173S^*) | Complementation of *ahpC^C173S^* in Δ*ahpC*, Str^r^, Tc^r^ | This study |
| Δ*ahpC*(*ahpC^C171SC173S^*) | Complementation of *ahpC^C171SC173S^* in Δ*ahpC*, Str^r^, Tc^r^ | This study |
| **Plasmids** |  |  |
| pME6032 | Shuttle vector, Tc^r^ | (Si et al., 2017) |
| pME6032-*ahpC* | *ahpC* under the control of chloramphenicol resistance gene promoter in plasmid pME6032, Tc^r^ | This study |
| pME6032*-ahpC^C57S^* | *ahpC^C57S^* under the control of chloramphenicol resistance gene promoter in plasmid pME6032, Tc^r^ | This study |
| pME6032-*ahpC^C171S^* | *ahpC^C171S^* under the control of chloramphenicol resistance gene promoter in plasmid pME6032, Tc^r^ | This study |
| pME6032-*ahpC^C173S^* | *ahpC^C173S^* under the control of chloramphenicol resistance gene promoter in plasmid pME6032, Tc^r^ | This study |
| pME6032-*ahpC^C171SC173S^* | *ahpC^C171SC173S^* under the control of chloramphenicol resistance gene promoter in plasmid pME6032, Tc^r^ | This study |
| pET-21a | Expression vector with C-terminal hexahistidine affinity tag, Amp^r^ | Novagen |
| pET-21a-*ahpD* | pET-21a carrying *ahpD* (*BTH_I2091*) coding region, Amp^r^ | This study |
| pET-21a-*ahpD^C131S^* | pET-21a carrying *ahpD^C131S^* coding region, Amp^r^ | This study |
| pET-21a-*ahpD^C134S^* | pET-21a carrying *ahpD^C134S^* coding region, Amp^r^ | This study |
| pET-28a | Expression vector with N-terminal hexahistidine affinity tag, Km^r^ | Novagen |
| pET-28a-*ahpC* | pET-28a carrying *ahpC* coding region, Km^r^ | This study |
| pET-28a-*ahpC^C57S^* | pET-28a carrying *ahpC^C57S^* coding region, Km^r^ | This study |
| pET-28a-*ahpC^C171S^* | pET-28a carrying *ahpC^C171S^* coding region, Km^r^ | This study |
| pET-28a-*ahpC^C173S^* | pET-28a carrying *ahpC^C173S^* coding region, Km^r^ | This study |
| pET-28a-*ahpC^C171SC173S^* | pET-28a carrying *ahpC^C171SC173S^* coding region, Km^r^ | This study |
| pET-28a-*ahpC^C57SC171S^* | pET-28a carrying *ahpC^C57SC171S^* coding region, Km^r^ | This study |
| pET-28a-*ahpC^C57SC173S^* | pET-28a carrying *ahpC^C57SC173S^* coding region, Km^r^ | This study |
| pET-28a-*lpd* | pET-28a carrying *lpd* coding region, Km^r^ | This study |
| pET-28a-*sucB* | pET-28a carrying *sucB* coding region, Km^r^ | This study |
| pET-28a-*trxB* | pET-28a carrying *trxB* coding region, Km^r^ | This study |
| pET-28a-*trxC* | pET-28a carrying *trxC* coding region, Km^r^ | This study |
| pGEX-6P-1 | Expression vector with N-terminal GST-tag, Amp^r^ | Novagen |
| pGEX-6P-1-*ahpC* | pGEX-6P-1 carrying *ahpC* coding region, Amp^r^ | This study |
| pDM4-phes | Suicide vector, mobRK2, oriR6K, pair, phes | (Si et al., 2017) |
| pDM4-phes-Δ*ahpC* | Construct used for in-frame deletion of *ahpC*, Cm^r^ | This study |

*Str^r^, Cm^r^, Km^r^, Tc^r^ and Amp^r^ represent resistance to [streptomycin](D:/%E5%B8%B8%E7%94%A8%E8%BD%AF%E4%BB%B6/Dict/7.1.0.0421/resultui/dict/?keyword=streptomycin), chloramphenicol, kanamycin, tetracycline and ampicillin at 100, 20, 50, 50 and 100 μg ml^-1^, respectively.

Table S2. Primers used in this study

| **Primers** | **Sequence（5′ to 3′）** | **Function** |
| --- | --- | --- |
| *ahpC*-F *Bam*HI | CGCGGATCCATGAAGACCGTGGGCGATAAA | To generate pET-28a-*ahpC,* pGEX-6P-1-*ahpC and* pME6032*-ahpC* |
| *ahpC-*R *Xho*I | CCGCTCGAGTTACAGCGAGCCGCCGAT |  |
| *ahpC-*M1F *Spe*I | CTAGACTAGTGAGCCTCGTGCACCAGCC | To generate pDM4-phes-Δ*ahpC* |
| *ahpC-*M1R | GTCCGTTTGCTTTTCTTCGTGGTTGTTGAA |  |
| *ahpC*-M2F | ACGAAGAAAAGCAAACGGACGAACTCTGAA |  |
| *ahpC*-M2R *Bgl*II | GGAAGATCTGTGAACGTGAAGGCGAAGAC |  |
| *ahpC^C57S^*-F | CTTCACGTTCGTCT*C*CCCGACGGAAATCG | To generate pET-28a-*ahpC^C57S^*  *and* pME6032*-ahpC^C57S^* |
| *ahpC^C57S^*-R | CGATTTCCGTCGGG*G*AGACGAACGTGAAG |  |
| *ahpC^C171S^*-F | CGGACGAACTCT*C*CCCGTGCAACCG | To generate pET-28a-*ahpC^C171S^ and*  pME6032*-ahpC^C171S^* |
| *ahpC^C171S^*-R | CGGTTGCACGGG*G*AGAGTTCGTCCG |  |
| *ahpC^C173S^*-F | ctgcaaacggacgaactctgcccgagcaaccgcgcgatcggcggc | To generate pET-28a-*ahpC^C173S^ and* pME6032*-ahpC^C173S^* |
| *ahpC^C173S^*-R | GCCGCCGATCGCGCGGTTGCTCGGGCAGAGTTCGTCCGTTTGCAG |  |
| *ahpC^C171SC173S^*-F | ctgcaaacggacgaactcagcccgagcaaccgcgcgatcggcggc | To generate pET-28a-*ahpC^C171SC173S^ and* pME6032*-ahpC^C171SC173S^* |
| *ahpC^C171SC173S^*-R | GCCGCCGATCGCGCGGTTGCTCGGGCTGAGTTCGTCCGTTTGCAG |  |
| *ahpD*-*Bam*HI-F | CGCGGATCCATGGAATTCATCGATTCGATTAAGG | To generate pET-21a-ahpD, pET-21a-*ahpD^C131S^* and pET-21a-*ahpD^C134S^* |
| *ahpD*-*Xho*I-R | CCGCTCGAGCTTGCCTTCTGCCGCGATCA |  |
| *ahpD^C131S^*-F | GTAAGAGCCACTTCTGCGTGAA | To generate pET-21a-*ahpD^C131S^* |
| *ahpD^C131S^*-R | TTCACGCAGAAGTGGCTCTTAC |  |
| *ahpD^C134S^*-F | GTAAGTGCCACTTCAGCGTGAA | To generate pET-21a-*ahpD^C134S^* |
| *ahpD^C134S^*-R | TTCACGCTGAAGTGGCACTTAC |  |
| *lpd*-F | CGGGATCCATGTCGAAGGAATTTGACGTCG | To generate pET-28a-*lpd* |
| *lpd*-R | ACGCGTCGACTTAGCTGTTGAGCGAGCGCTTG |  |
| *sucB*-F | CGGGATCCATGAGTCAAGCGATCGAAGTC | To generate pET-28a-*sucB* |
| *sucB*-R | ACGCGTCGACTCAAAGAATGATGCGGCGG |  |
| *trxB*-F | CGGGATCCATGTCCACGCCCAAACACGCG | To generate pET-28a-*trxB* |
| *trxB*-R | ACGCGTCGACTTACTTGTGGTCGTGCAGGC |  |
| *trxC*-F | CGGGATCCATGCAGCGCTCACGCCCTTC | To generate pET-28a-*trxC* |
| *trxC*-R | CCCAAGCTTTTAGAGATGGCTGTCCAGGAAC |  |
| P*_ahpC_-*F | GTTTCCGAGCGCGAAAGCG | To produce the 231 bp EMSA *ahpC* promoter DNA |
| P*_ahpC_-*R | CGGATGCTCCTGTGTAAGTTG |  |
| Control-F | CGAGACCGTCACCGAAGCG | To produce the 230 bp EMSA control DNA of AhpC |
| Control-R | GTGCTCACGGCGCCATGCGAG |  |

Underlined sites indicate restriction enzyme cutting sites added for cloning.

Si, M., Zhao, C., Burkinshaw, B., Zhang, B., Wei, D., Wang, Y., Dong, T. G., Shen, X. (2017) Manganese scavenging and oxidative stress response mediated by type VI secretion system in *Burkholderia thailandensis*. *Proc. Natl. Acad. Sci. U S A*, 114(11):E2233-E2242.
